# Ostreid Herpesvirus-1 Infects Specific Hemocytes in Ark Clam, *Scapharca broughtonii*

**DOI:** 10.3390/v10100529

**Published:** 2018-09-28

**Authors:** Lusheng Xin, Chen Li, Changming Bai, Chongming Wang

**Affiliations:** 1 Qingdao Key Laboratory of Mariculture Epidemiology and Biosecurity, Key Laboratory of Maricultural Organism Disease Control, Ministry of Agriculture, Yellow Sea Fisheries Research Institute, Chinese Academy of Fishery Sciences, Qingdao 266071, China; xinls@ysfri.ac.cn (L.X.); lichen@ysfri.ac.cn (C.L.); baicm@ysfri.ac.cn (C.B.); 2 Function Laboratory for Marine Fisheries Science and Food Production Processes, Qingdao National Laboratory for Marine Science and Technology, Qingdao 266071, China

**Keywords:** ostreid herpesvirus 1, hemocytes, apoptosis, OsHV-1 infection, *Scapharca broughtonii*

## Abstract

High levels of ostreid herpesvirus 1 (OsHV-1) were detected in hemocytes of OsHV-1 infected mollusks. Mollusk hemocytes are comprised of different cell types with morphological and functional heterogeneity. Granular cells are considered the main immunocompetent hemocytes. This study aimed to ascertain if OsHV-1 infects specific types of hemocytes in ark clams. Types of hemocytes were first characterized through microexamination and flow cytometry. In addition to a large group of red cells, there were three types of recognizable granular cells in ark clams. Type II granular cells were mostly found with OsHV-1 infection by transmission electron microscope (TEM) examination, and represented the hemocyte type that was susceptible to OsHV-1 infection. The subcellular location of OsHV-1 particles in apoptotic type II granular cells was further analyzed. Some OsHV-1 particles were free inside the apoptotic cells, which may contribute to OsHV-1 transmission among cells in the host, some particles were also found enclosed inside apoptotic bodies. Apoptosis is an important part of the host defense system, but might also be hijacked by OsHV-1 as a strategy to escape host immune attack. Following this investigation, a primary culture of type II granular cells with OsHV-1 infection would facilitate the research on the interaction between OsHV-1 and mollusk hosts.

## 1. Introduction

Herpes-type viruses were first reported in the diseased oyster *Crassostrea virginica* in 1972 [1]. After that, summer mass mortalities of molluscs have been detected with the herpesvirus infection. Nearly total mortality of *Crassostrea gigas*, *Ostrea edulis* and *Ruditapes philippinarum* larvae had been reported with herpesvirus infection in French hatcheries since the 1990s [2,3,4]. The herpesvirus was subsequently isolated from infected *C. gigas* larvae, then was sequenced and classified as a member of the herpesviridae under the name ostreid herpesvirus 1 (OsHV-1) [5]. Until recently, OsHV-1 infection associated mass mortalities of mollusk constantly arose [6,7,8]. OsHV-1 is considered a major pathogen of shellfish. The overall structure of OsHV-1 capsid is similar to those of other previously reported herpesviruses with a highly ordered icosahedral-shape nucleocapsid of about 120 nm in diameter [5]. The nucleocapsid is enclosed within the envelope, a polymorphic lipid bilayer. Typical structural features of OsHV-1 facilitate effective clinical diagnosis of OsHV-1 infection by transmission electron microscope (TEM) [9,10].

Although OsHV-1 infection commonly leads to mass mortalities in mollusks, the innate immune system of the host does act against the infecting OsHV-1 [11,12], especially cellular immune strategies, including autophagy and apoptosis. A reduction in OsHV-1 DNA was found when autophagy was stimulated by the administration of carbamazepine or starvation, suggesting that viral particles might be cleared when autophagy is induced [13]. It was also revealed that the trigger factors (caspases), as well as inhibitors of apoptosis proteins (IAPs) in hosts were both up-regulated in response to OsHV-1 infection. Meanwhile, IAP homologs containing the BIR domain could also be coded by OsHV-1 which might further interfere with the host regulation of apoptosis [14,15]. This would imply that apoptosis is a key part of antiviral response and under complex or antagonistic control [11]. As the major executors of cellular immune defense [16,17], hemocytes are attacked by OsHV-1, and an increased level of OsHV-1 DNA has been detected in mollusk hemocytes post infection [18,19]. Similar to crustaceans, at least three types of mollusk hemocytes have been isolated according to their size and granularity, including agranulocytes (hyaline cells), semi-granular and granular cells [20,21]. Among them, semi-granular and granular cells were found as two specific host hemocyte types targeted by white syndrome spot virus (WSSV) in shrimp [20]. However, whether OsHV-1 also targets specific types of mollusk hemocytes is unknown.

Besides oyster and scallop, ark clam, *Scapharca broughtonii*, has emerged as a new host for OsHV-1. OsHV-1 infection was first detected in diseased ark clams along the coast of Northern China in the early summer of 2012 [6,22]. Compared with oyster and scallop hemocytes, the composition of hemocyte types of ark clams seems more complex according to hemocyte morphological features [23], and red cells exist in ark clams but neither in oysters nor scallops. In this study, whether OsHV-1 targeted specific types of hemoyctes in ark clams was analyzed. Firstly, the hemocyte types of ark clams were characterized through morphological observation and flow cytometry. Then hemocyte types with OsHV-1 infection were further identified by TEM examination. Hemocytes with different stages of OsHV-1 infection were further scanned, and we assessed the subcellular localization of OsHV-1 particles in apoptotic hemocytes, to facilitate a better understanding of host cellular apoptosis response to OsHV-1 infection.

## 2. Materials and Methods

### 2.1. Ark Clams

Healthy ark clams (two years old, average shell length of 55 mm) were collected from a local shellfish farm in Qingdao, China. These ark clams were firstly acclimated with aerated sea water at 18.0 °C for two weeks in our laboratory, then were randomly selected for the following assays. Relevant experiments were approved by the local animal care and use committee, and conducted according to the regulations of local and central government.

### 2.2. Experimental OsHV-1 Infection and Sample Collection

Experimental OsHV-1 infection was adopted through injection of OsHV-1 inoculum with modification [24,25]. Briefly, OsHV-1 inoculum was firstly prepared using filtered (0.22 μm) mantle tissue homogenate (FMH) of OsHV-1 naturally infected in the field ark clams (stored at −80 °C). Then OsHV-1 concentration of the inoculum was quantified through DNA extraction and RT-qPCR as described below. One hundred ark clams were randomly separated into two groups. One group was injected with 10^5^ OsHV-1 copies per ark clam in a total volume of 100 μL FMH, while the other group was injected with the same volume of FMH from healthy ark clams as the control.

For the OsHV-1 quantification assay, each ark clam tissue sample was collected from three ark clams and mixed together at 72 h post-injection. Each tissue sample was set with triple replicates. Then collected tissue samples were directly stored at −80 °C for the following assay. 

### 2.3. Quantification of OsHV-1 Load by RT-qPCR

Total DNA of each ark clam tissue sample was extracted using the DNeasy Blood and Tissue kit (Qiagen, Hilden, Germany) according to the manual. The concentration of extracted DNA was detected by spectrophotometer NanoDrop2000 (Thermo-Scientific, Waltham, MA, USA).

The quantification of OsHV-1 load was performed by RT-qPCR as previously described, targeting a DNA polymerase catalytic subunit of OsHV-1 [26]. Briefly, a 25 μL reaction system consisted of 12.5 μL of 2× FastStart Essential DNA Probes Master (Roche Diagnostics, Risch-Rotkreuz, Switzerland), 1.0 μL of primer BF (10 μM) (5′-GTCGCATCTTTGGATTTAACAA-3′), 1.0 μL of primer B4 (10 μM) (5′-ACTGGGATCCGACTGACAAC-3′), 0.5 μL of TaqMan^®^ probes (10 μM) (5′-FAM-TGCCCCTGTCATCTTGAGGTATAGACAATC-BHQ-3′), 2 μL of template DNA and 8 μL ddH_2_O. The reaction was started with 1 cycle of 95 °C for 10 min, followed with 40 cycles of 95 °C for 10 s, 60 °C for 30 s based on Bio-Rad CFX Connect RealTime system (Bio-Rad Laboratories, Hercules, CA, USA). The OsHV-1 load was finally calculated according to the standard curve, which was created from a 10-fold dilution series (10^7^–10^1^ copies μL^−1^) of plasmids containing the target sequence. Data was expressed in OsHV-1 copy number per ng total tissue DNA for the three replicates.

### 2.4. Flow Cytometry Analysis

Hemolymph was randomly pooled from five healthy ark clams with anticoagulation (260 U/mL heparin sodium). Then hemocytes were pre-filtered through a 50 μm mesh gauze and suspended in filtered (0.22 μm) sea water as one sample. Hemocytes were sorted and counted based on their cellular size and granularity by a CyFlow Space flow cytometry. Three repeated samples were respectively detected. Data were expressed from 10,000 counts for each sample.

### 2.5. Hemocyte Morphologic Observation

Extracted hemolymph from healthy ark clams was left standing for 30 min to stratify at 18.0 °C, before hemocytes in three formed layers were, respectively, transferred to 48-well plates and observed under light microscopy. Then, hemocytes on plates were fixed with 4% paraformaldehyde for 10 min. DAPI was used to stain nuclei for 5 min followed by PBS washing, and the hemocytes were characterized by a laser scanning confocal microscopy (LSCM) (Nikon A1, Tokyo, Japan).

### 2.6. Transmission Electron Microscopy (TEM) Examination

Hemocyte, hepatopancreas and mantle were collected from nine individual ark clams with OsHV-1 inoculation at 72 h, then were fixed with 2.5% glutaraldehyde (pH 7.2) in 0.2 M sodium cacodylate for 24 h at 4 °C. After further fixation with 1% osmium tetroxide, tissue samples were dehydrated and embedded in Spurr’s resin. Ultrathin sections were prepared using Ultracut-E ultramicrotome, then stained with uranyl acetate (pH 6.0), and examined with a JEOL JEM-1200 electron microscope (Jeol Solutions for Innovation, Peabody, MA, USA) at an accelerating voltage of 80–100 kV. Hemocyte, hepatopancreas and mantle from nine healthy individual ark clams were treated the same as above.

## 3. Results

### 3.1. OsHV-1 Load in Different Ark Clam Tissues

**Figure 1:**
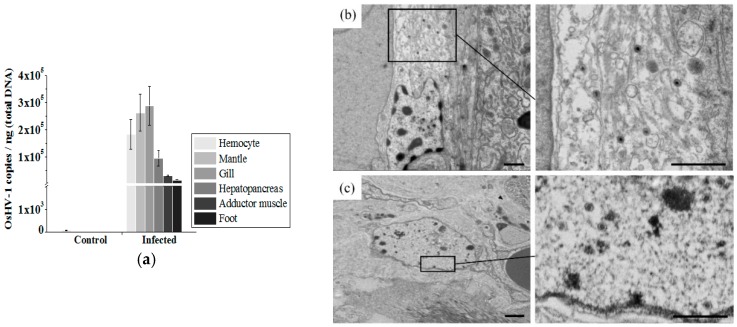
Ostreid herpesvirus 1 (OsHV-1) load in different tissues of infected ark clams. (**a**) RT-qPCR quantification of OsHV-1 load in ark clam tissues post 72 h of infection. (**b**) Transmission electron microscope (TEM) examination in infected ark clam hepatopancreas, and (**c**) mantle tissue. (Scale bar = 1 μm).

The OsHV-1 loads in different ark clam tissues were quantified by RT-qPCR post 72 h of OsHV-1 inoculation. Compared with the control group (injected with FMH of healthy ark clams), significant increase of OsHV-1 loads could be detected in all tested tissues including hemocytes, mantle, gill, hepatopancrease, and adductor muscle and foot. OsHV-1 load was up to more than 10^4^ copies per ng total DNA in each test tissue samples (Figure 1a). The presence of OsHV-1 particles in the hemocytes, hepatopancrease and mantle of infected ark clams was further examined by TEM. In addition to in hemocytes (Figures 4 and 6), the intact OsHV-1 particles could be found inside the cells of the hepatopancrease (Figure 1b) and mantle (Figure 1c), characterized by a typical nucleocapsid enclosed inside an envelope with an estimated diameter of 120 nm.

### 3.2. Characterization of Different Types of Ark Clam Hemocytes

**Figure 2:**
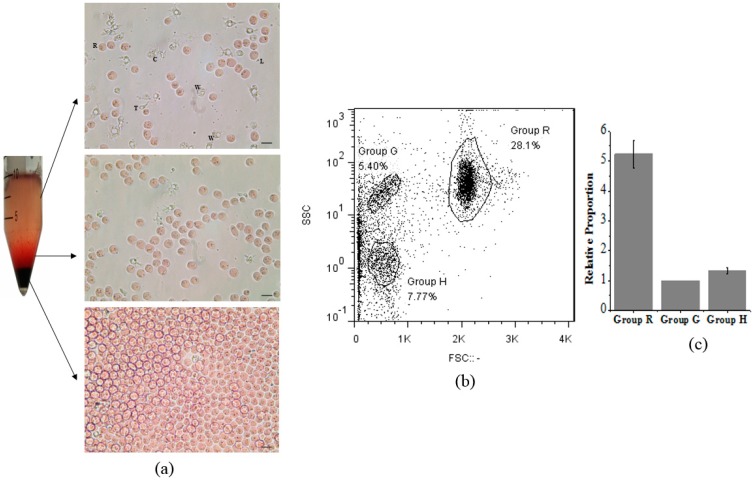
Isolation of different types of ark clam hemocytes. (**a**) Ark clam hemocytes were subjected to stratification and microscopy examination. (Scale bar = 20 μm). Red cells, R; White cells, W; Thrombus cells, T; Clotting cells, C; Lymphocytes, L. (**b**) Analysis of different hemocyte groups by flow cytometry. Group R denotes red cells, Group G denotes white cells, Group H denotes thrombus and lymph cells. (**c**) The histogram shows the relative proportions of different hemocyte groups.

Ark clam hemocytes could be roughly separated into three layers after standing for 30 min (Figure 2a). Red cells were mainly on the bottom, while other types of hemocytes were mainly found on the top, including white cells, clotting cells, thrombus cells and lymphocytes. Three recognizable hemocyte groups were shown by the flow cytometric assay (Figure 2b), which was in accordance with previous studies [23]. Group R denoted red cells, Group G denoted white cells, Group H denoted thrombus and lymph cells. The amount of Group R presented about five-fold that of Group G or Group H (Figure 2c).

**Figure 3:**
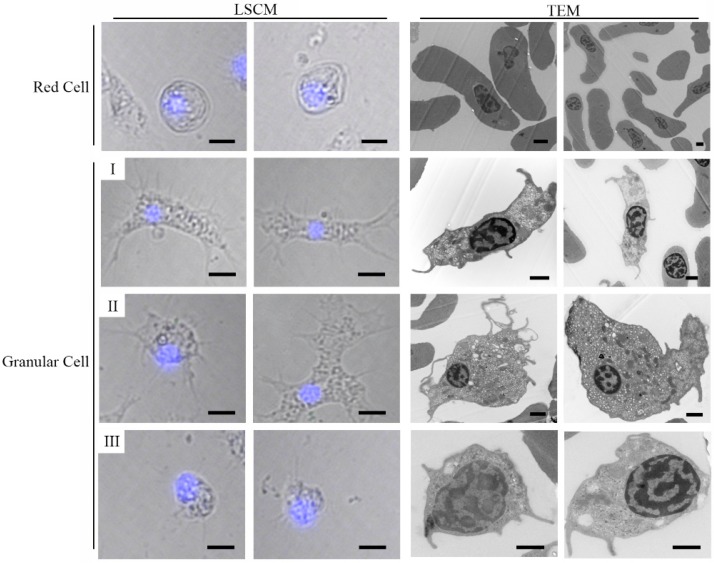
Examination of different ark clam hemocytes by TEM (Scale bar = 1 μm) and laser scanning confocal microscopy (LSCM) (Scale bar = 10 μm), nucleus stained with DAPI (blue).

According to the results of LSCM and TEM, at least three types (I, II and III) of granular cells could be found (Figure 3). Type I presented a strip cell, corresponding to a thrombus cell; type II was characterized with a small nucleo-cytoplasmic ratio and malleable shape, corresponding to a white cell; type III showed a large nucleo-cytoplasmic ratio, corresponding to a lymph cell. All three types of granular cells could stretch out dendrites. Different to granular cells, red cells presented a round pie shape. Few organelles, apart from the nuclei, could be found by TEM.

### 3.3. Identification of OsHV-1 Infected Hemocytes by TEM

**Figure 4:**
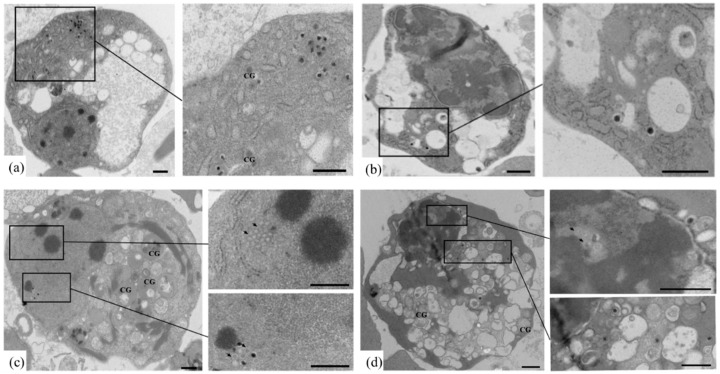
TEM examination of OsHV-1 infected ark clam hemocytes. (**a**–**d**), OsHV-1 infected hemocytes. Arrows show assembling OsHV-1 particles in nuclear region. CG, cytosolic granules. (Scale bar = 500 nm).

**Figure 5:**
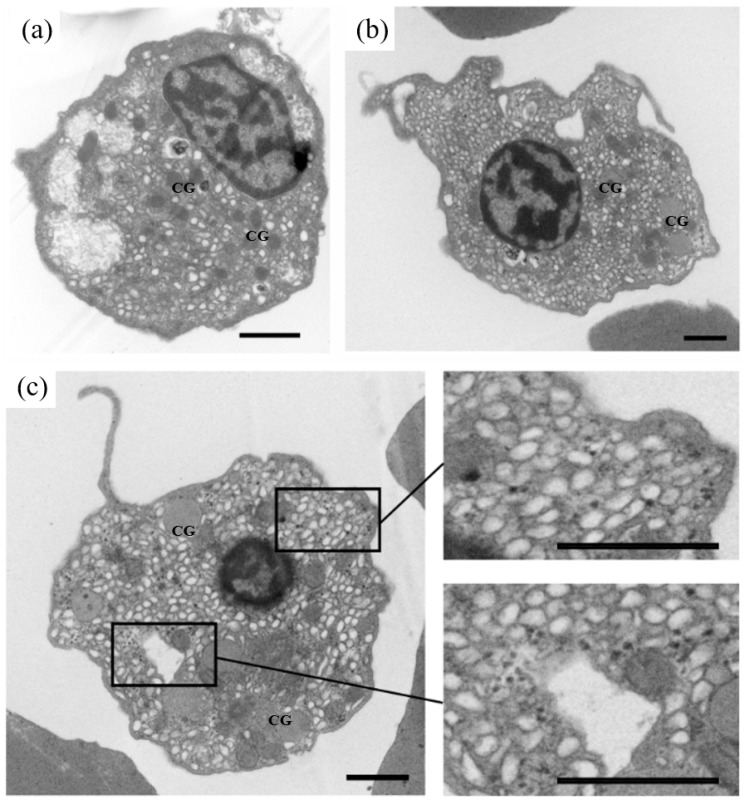
Characteristics of ark clam hemocytes that were susceptible to OsHV-1 infection. (**a**–**c**) healthy hemocytes without OsHV-1 infection. CG, cytosolic granules. (Scale bar = 1 μm).

According to the TEM assay, no OsHV-1 infected red cells were found in OsHV-1 infected ark clams. Type II granular cells (white cells) with assembling OsHV-1 capsids in nuclear regions were found, but neither type I nor type III cells with assembling OsHV-1 capsids were found (Figure 4). Figure 5 showed the OsHV-1 susceptible type II granular cells with a small nucleo-cytoplasmic ratio from healthy ark clams; comparatively, there were no OsHV-1 particles. 

**Figure 6:**
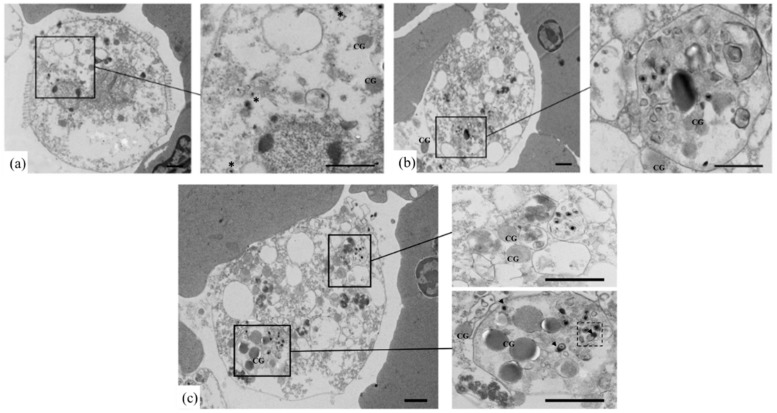
The apoptosis of OsHV-1 infected ark clam hemocytes. (**a**–**c**), OsHV-1 infected hemocytes. Free OsHV-1 particles (indicated by asterisks); OsHV-1 particles in apoptosis body (indicated by arrows); disintegrated OsHV-1 particles (as shown in the dotted box). CG, cytosolic granules. (Scale bar = 1 μm).

Type II granular cells (white cells) with assembling OsHV-1 capsids in the nuclear regions indicated the cells were mid-infection, with nuclear enlargement and disintegration. The cellular apoptosis is shown in Figure 6, indicating a different stage of OsHV-1 infection. Some OsHV-1 particles were enclosed inside the apoptosis bodies, and several disintegrated ones could be found, as shown in the dotted box (Figure 6).

## 4. Discussion

Mollusks rely on an innate immune system to protect themselves from invading environmental microbes. Hemocytes comprise an important aspect of their innate immune system, especially granular cells [27], which are involved in both cellular and humoral immunity, and serve as an immune modulator through a neuroendocrine-immune (NEI) regulatory network [16]. However, hemocytes carry a high level of OsHV-1 load in infected mollusks, which might directly disturb the whole innate immune system of mollusks and lead to death.

Besides hemocytes, other tissues also presented high levels of OsHV-1 load. Despite no tissue specificity, OsHV-1 infection showed cellular specificity in hemocytes. Three types of mollusk hemocytes have been reported according to hemocyte size and granularity [21,27,28], including agranulocytes, semigranulocytes and granulocytes. Despite the hemocyte types in ark clams seeming more complex, there were still three groups of hemocytes seen, as in other mollusks. However, the three groups (Group R denoting red cells, Group G denoting white cells, and Group H denoting thrombus and lymph cells) in ark clams were not in accordance with the previously reported agranulocytes, semigranulocytes and granulocytes as seen in other mollusks. Group R was the largest group of hemocytes in ark clams. In addition to red cells, three types of granular cells were distributed in the other two groups (Type II (white cells) in Group G, and Types I (thrombus cells) and II (lymph cells) in Group H). Type II granular cells were mostly found with OsHV-1 infection and represented the susceptible hemocyte type to OsHV-1 infection. They were previously described as white cells that showed strong phagocytosis [23]. Meanwhile, phagocytosis was previously considered as a method of OsHV-1 invading into cells [11], which might greatly contribute as to why type II granular cells are vulnerable to OsHV-1 infection.

The nucleocapsids of OsHV-1 are assembled in the nuclear regions of type II granular cells in ark clams. Apoptosis of type II granular cells was triggered post OsHV-1 infection. According to gene expression level [11], it is hard to identify whether the apoptosis of the host cell is active or passive. Nevertheless, it was found that some OsHV-1 particles were enclosed into apoptotic bodies and might be finally destroyed and cleared, while others were free in the apoptotic host cells according to TEM examination. Apoptosis of type II granular cells could not thoroughly clear OsHV-1 particles, contrarily, that would contribute to further diffusion of mature OsHV-1 particles (Figures S1 and S2). With apoptosis of type II granular cells, host immune defense systems should be weakened and disturbed. Thus apoptosis is proposed to be a major immune escape mechanism of OsHV-1, which is similar to that of human immunodeficiency virus (HIV), promoting apoptosis of host immune-related cells, then leading to the destruction of immune effectors to evade the host immune system attack [29].

## 5. Conclusions

In conclusion, our studies suggest that the hemocytes, especially type II granular cells (white cells), are susceptible to OsHV-1 infection. Apoptosis of type II granular cells could not effectively eliminate OsHV-1 particles, which is proposed as a mechanism to facilitate OsHV-1 particle transmission among cells in the host. Meanwhile the host immune defense system would be disturbed from the apoptosis of the immune modulator cells. Apoptosis is considered as an OsHV-1 strategy to escape host immune attack.

## Supplementary Materials

The following are available online at http://www.mdpi.com/1999-4915/10/10/529/s1, Figure S1: The apoptosis of OsHV-1 infected ark clam hemocytes, Figure S2: The apoptotic bodies of OsHV-1 infected ark clam hemocytes.
