# Supplementary material for: Ostreid Herpesvirus-1 Infects Specific Hemocytes in Ark Clam, Scapharca broughtonii
Source: Viruses. 2018 Sep 28;10(10):529. doi: 10.3390/v10100529 (PMC6213218; doi:10.3390/v10100529)
Supplement: Supplementary file 1 [file viruses-10-00529-s001.pdf]

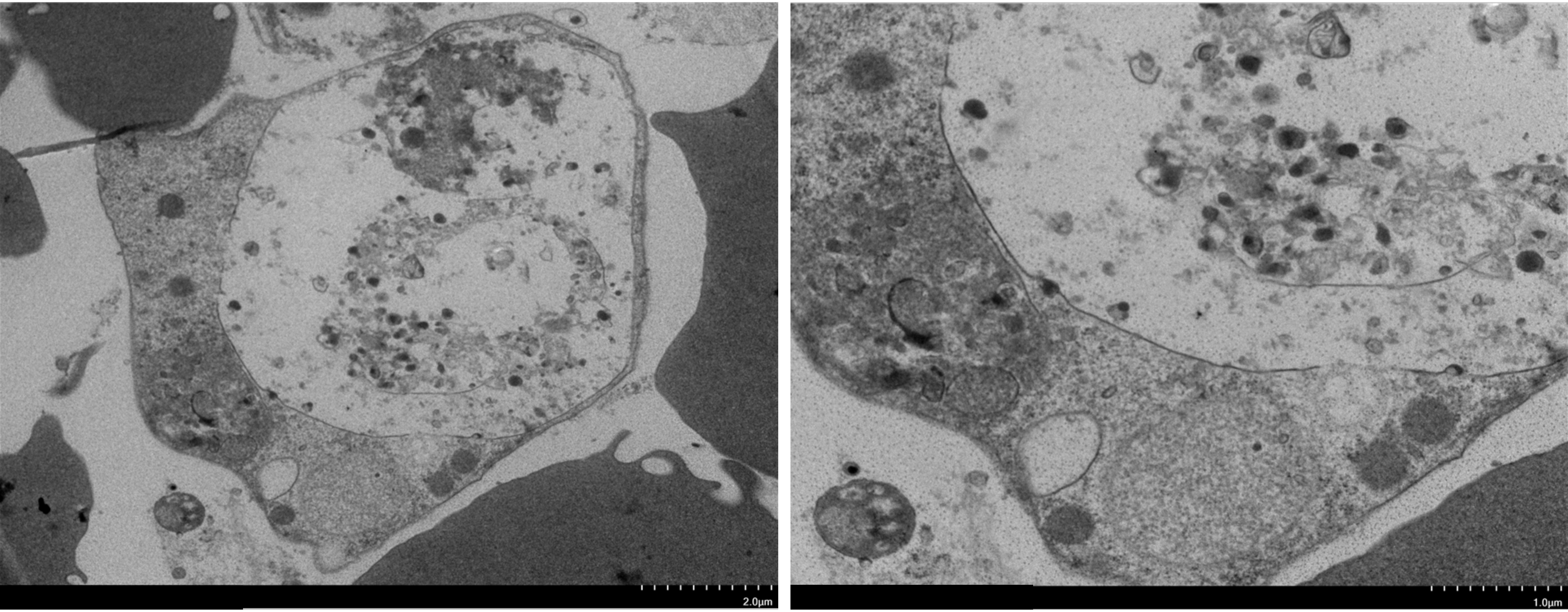

Figure S1. The apoptosis of OsHV-1 infected ark clam hemocytes.

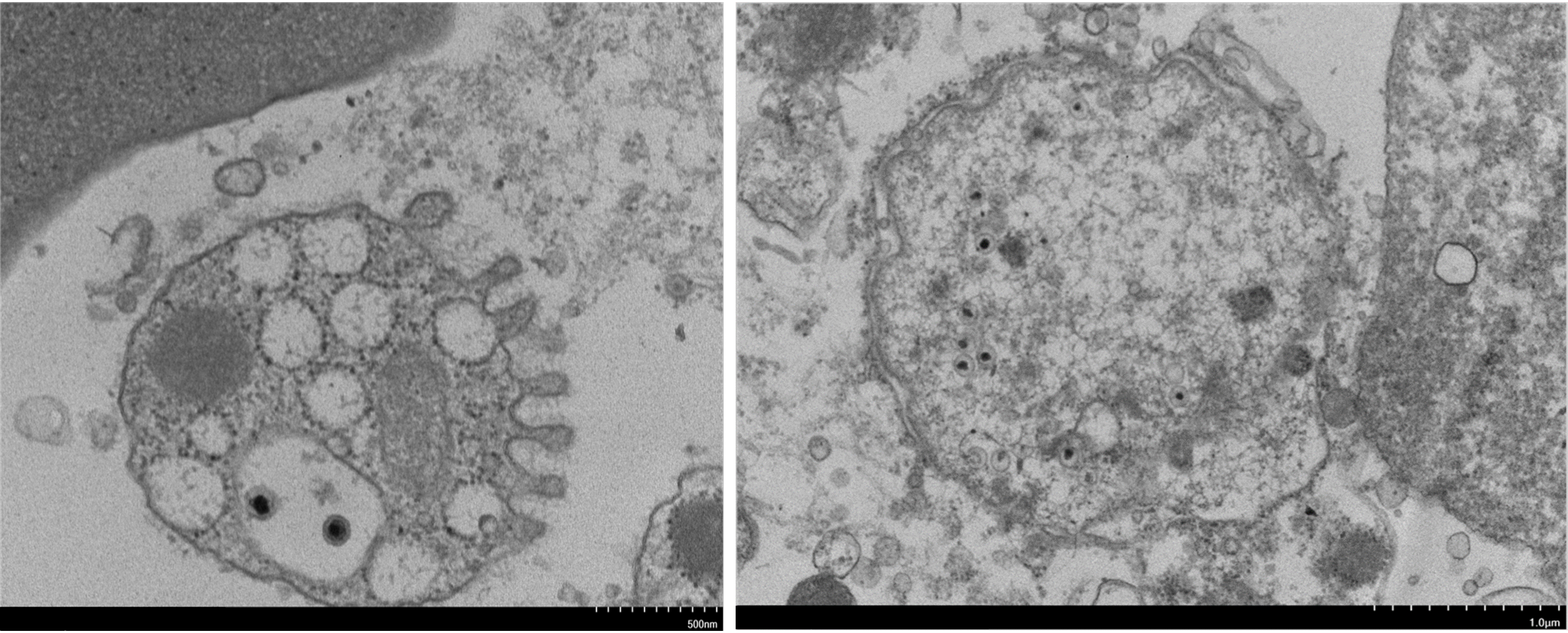

Figure S2. The apoptotic bodies of OsHV-1 infected ark clam hemocytes.
